# Immobilization of BoPAL3 Phenylalanine Ammonia-Lyase on Electrospun Nanofibrous Membranes of Polyvinyl Alcohol/Nylon 6/Chitosan Crosslinked with Dextran Polyaldehyde

**DOI:** 10.3390/polym15183699

**Published:** 2023-09-08

**Authors:** Chun-Yen Hsieh, Pei-Yu Hong, Lu-Sheng Hsieh

**Affiliations:** 1 Department of Pathology and Laboratory Medicine, Shin Kong Wu Ho-Su Memorial Hospital, Taipei City 111, Taiwan; t012874@ms.skh.org.tw; 2 Department of Food Science, College of Agriculture and Health, Tunghai University, Taichung 40704, Taiwan; elaine860125@gmil.com

**Keywords:** *Bambusa oldhamii*, chitosan (CS), dextran polyaldehyde, electrospun nanofiber, enzyme kinetics, nylon 6, phenylalanine ammonia-lyase (PAL), polyvinyl alcohol (PVA)

## Abstract

Phenylalanine ammonia-lyase (PAL, EC 4.3.1.24) is common in plants and catalyzes the formation of *trans*-cinnamic acid and ammonia via phenylalanine deamination. Recombinant *Bambusa oldhamii* BoPAL3 protein expressed in *Escherichia coli* was immobilized on an electrospun nanofibrous membrane using dextran polyaldehyde as a crosslinker. The immobilized BoPAL3 protein exhibited comparable kinetic properties with the free BoPAL3 protein and could be recycled for six consecutive cycles compared with the free BoPAL3 protein. The residual activity of the immobilized BoPAL3 protein was 84% after 30 days of storage at 4 °C, whereas the free BoPAL3 protein retained 56% residual activity in the same storage conditions. Furthermore, the resistance of the immobilized BoPAL3 protein to chemical denaturants was greatly increased. Therefore, the BoPAL3 protein can be immobilized using the natural dextran polyaldehyde crosslinker in place of the conventional chemical crosslinker. Nanofibrous membranes made from polyvinyl alcohol (PVA), nylon 6, and chitosan (CS) are incredibly stable and useful for future industrial applications.

## 1. Introduction

**Figure 1:**
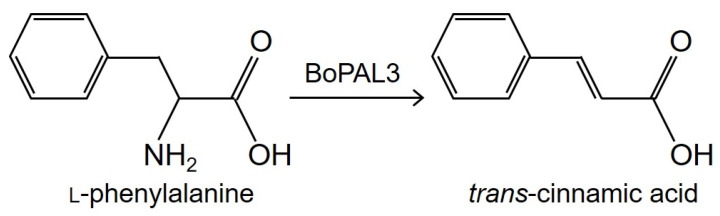
BoPAL3 enzyme catalyzes the deaminated conversion of L-phenylalanine to *trans*-cinnamic acid.

As one of the well-researched enzymes in all plants, phenylalanine ammonia-lyase (PAL, EC 4.3.1.24, renamed from EC 4.3.1.5), catalyzes the non-oxidative deamination process of phenylalanine to produce *trans*-cinnamic acid and ammonia (Figure 1) [1,2,3]. PAL proteins are generally encoded by multiple gene families in many plants, such as *Arabidopsis thaliana* [4,5], *Bambusa oldhamii* [3,6,7,8,9,10], and *Nicotiana tabacum* [2]. PAL activity is detectable in nearly all plants as well as in cyanobacteria [11], some fungi [12], and limited microorganisms, such as *Photorhabdus luminescens* [13]. Cinnamic acid is used as the starting precursor for thousands of phenylpropanoid compounds, including anthocyanins, coumarins, flavonoids, lignins, and so on [14,15,16]. The benefits of *trans*-cinnamic acid for human health are widely acknowledged, and these benefits include anti-aging, anti-cancer, anti-diabetes, anti-fungal, and even anti-inflammatory effects [17,18,19,20]. Thus, there is a possibility of using *trans*-cinnamic acid as a therapeutic agent for many human diseases, such as Alzheimer’s disease, tuberculosis, and viral infections [21]. Indeed, *trans*-cinnamic acid, according to recent studies, is a potential chemical that can help to reduce obesity in animal models [22,23]. Furthermore, cinnamic acid derivatives are frequently utilized as scents and UV-blocking chemicals in the cosmetic sector [24] and also have anti-bacterial, anti-cancer, and antioxidant properties [25,26,27,28].

Substrate specificity by the phenylalanine ammonia-lyase enzyme is deeply studied in *Bambusa oldhamii* [3,6,7,8,9,10]. Four *BoPAL* genes are heterologously expressed in *Escherichia coli* or *Pichia pastoris*, and all recombinant proteins exhibit undisputed PAL activity [3]. The active site of all four BoPAL enzymes is composed of a conserved Ala-Ser-Gly tripeptide, which is converted into the 4-methylididene-imidazole-5-one (MIO) cofactor [7,8,9,10]. Recombinant BoPAL1 protein is highly specific to phenylalanine as are many PAL proteins from dicot plants [8,9]. Recombinant BoPAL2 and BoPAL3 proteins show minimal tyrosine ammonia-lyase (TAL) activity when using tyrosine as the substrate to produce p-coumaric acid and ammonia [3,7]. Recombinant BoPAL4 protein can not only use phenylalanine and tyrosine as substrates but also uses 3,4-dihydroxyphenylalanine (DOPA) as a substrate to yield caffeic acid and ammonia [10]. The His-123 in the BoPAL4 protein is the major site of substrate selectivity for tyrosine, and the recombinant BoPAL4-H123F mutant exhibits reduced TAL activity and enhanced PAL activity [10]. The specific TAL activity of the BoPAL3 protein is approximately a quarter that of the BoPAL4 protein [3]. The substrate selectivity site in the BoPAL3 protein is Phe-134, which is the same as in the BoPAL2 protein, although more studies should be conducted to investigate more substrate selectivity sites among these enzymes.

Enzymes are highly specific biocatalysts in nature that generally operate under mild reaction conditions, instead of chemical synthesis; however, free-form enzymes are more delicate and sensitive to temperature extremes, pH, and the presence of chaotropic chemical substances [29,30]. From a commercial viability point of view, these biocatalysts have low operational stability and difficulty recycling, resulting in high prices and low application values. In order to overcome the above limitation for enzymes in free form, the enzyme immobilization technique now offers a useful tool to improve enzyme performance in industrial applications [31,32,33,34]. Functional nanomaterials, such as nanofibers, nanoparticles, and nanocomposites, have drawn interest and attention because of their distinctive characteristics and intriguing uses in enzyme immobilization techniques [35,36,37]. Owing to their high surface-to-volume ratio, nanofibrous membranes can offer a sizable area for protein immobilization [36,38]. The electrospinning technique is a convincing method that has emerged in recent years that uses an electrostatic force to transform polymer solutions into nanofibrous membranes [38,39,40]. Previous studies have shown that immobilized enzymes have increased the resistance to extreme pH and temperature [41], increased reusability [42], extended storage stability, enhanced enzymatic activities [38,43], and so on.

Several studies have used a whole-cell conversion strategy to produce *trans*-cinnamic acid in *Escherichia coli* [44] and *Corynebacterium glutamicum* [45]. In 2021, *Petroselinum crispum* PAL protein was first entrapped on a polylactic acid matrix using the emulsion electrospinning technique [46]. In this present work, we utilized another membrane material to immobilize the BoPAL3 protein using the electrospinning technique to produce *trans*-cinnamic acid. We first cloned the cDNA and constructed a heterologous expression system to produce the *Bambusa oldhamii* recombinant BoPAL3 protein [3]. Polyvinyl alcohol (PVA) and nylon 6 polymers were selected as support materials for the BoPAL3 enzyme due to their high biocompatibility and good structural stability in aqueous solutions [38]. In this work, we created PVA/nylon 6 fibers with the help of chitosan (CS), which gives the fibers amino groups. By using the periodate-mediated oxidation method to produce dextran polyaldehyde, two reactive aldehyde groups were created that might be employed as a natural crosslinker for immobilizing the BoPAL3 enzyme onto a nanofiber membrane, which is safe, biodegradable, and environmentally friendly. PAL enzymatic activity, pH, temperature, kinetic parameters (*k*_cat_ and *K*_m_), storage stability, and chemical stability of the free and immobilized BoPAL3 proteins were studied.

## 2. Materials and Methods

### 2.1. Chemicals

Chitosan (low molecular weight (MW), 50–190 kDa), Coomassie Brilliant Blue R-250 (CBR) dye, dextran (MW 200 kDa), nylon 6, phenylalanine, polyvinyl alcohol (PVA, MW 85 kDa–124 kDa), and *trans*-cinnamic acid were purchased from MilliporeSigma, Burlington, MA, USA. Ampicillin, isopropyl β-D-thiogalactopyranoside (IPTG), and Luria–Bertani (LB) medium were obtained from Cyrusbioscience Inc., New Taipei City, Taiwan. Bio-Rad (Hercules, CA, USA) was the supplier of protein assay dye reagents, precision plus protein^TM^ dual colors standards, and chemical reagents for protein electrophoresis. Other chemicals used in this study were regent- or ACS-grade.

### 2.2. Expressions of Recombinant BoPAL3 Protein in Escherichia coli

The TOP10 strain of *E. coli* was used to propagate and overexpress the pTrcHis-BoPAL3 plasmid [3]. A total of 250 mL LB media (0.5% yeast extract, 1% tryptone, and 1% NaCl) and 100 μg/mL of ampicillin were used to inoculate the cells, which were, then, shaken at 200 rpm at 37 °C. When an OD_600_ reached 1.0 from 0.6, a final IPTG dose of 1 mM was added to induce the BoPAL3 protein expression, and then, transferred to a 30 °C shaker for a further 6–8 h incubation [47,48,49]. Centrifugation at 6000× *g* for 20 min was used to harvest the *E. coli* cells, and the pellets were kept at −20 °C, in a freezer, until use.

### 2.3. Preparation of Recombinant BoPAL3 Protein

Recombinant BoPAL3 protein was affinity purified on a Ni-NTA resin (Cyrus Bioscience Inc., New Taipei City, Taiwan) and chelated with nickel (Ni^2+^) ions. *E. coli* cells stored at −20 °C were resuspended in 20 mL lysis buffer containing 50 mM Tris–HCl (pH 7.5), 10 mM imidazole, 100 mM NaCl, and the indicated amount of protease inhibitors [3]. An Ultrasonic Processors Sonicator 3000 (Misonix, Farmingdale, NY, USA) was used to sonicate cells, which mechanically disrupts the cells. The cell lysate was centrifuged at 10,000× *g* for 10 min. A Ni-NTA column was used to apply the supernatant, and buffers containing either 50, 125, 250, or 500 mM of imidazole were used to competitively elute the recombinant BoPAL3 protein. The purity of eluted protein was estimated using polyacrylamide gel electrophoresis.

### 2.4. Sodium Dodecyl Sulfate Polyacrylamide Gel Electrophoresis

Sodium dodecyl sulfate polyacrylamide gel electrophoresis (SDS-PAGE) was carried out based on the previously described method [3,38]. Gels were stained with Coomassie Brilliant Blue R-250 dye after electrophoresis for 30 min, and then, de-stained using 20% methanol. Gel images were taken using a Gel Doc XR+ Imaging System (Bio-Rad, Hercules, CA, USA).

### 2.5. Preparation of Nanofibrous Membrane by Electrospinning Method

Polyvinyl alcohol (PVA), nylon 6, and chitosan (CS) polymer solutions were prepared as 4% (*w*/*v*), 8% (*w*/*v*), and 1% (*w*/*v*), respectively [38]. Three polymer mixes were combined and pushed into a spinneret using a 5 mL syringe with a 21 G/13 mm diameter, which was operated by an NE-300 pump (New Era Pump Systems, Farmingdale, NY, USA). The spinneret needle was charged with a voltage of 13.8 kV, using a high voltage power supply, Model FES-COS (Falco, New Taipei City, Taiwan). The needle tip and collector plate were 13 cm away. Then, the roller collector covered in baking paper was used to format electrospun PVA/nylon 6/CS nanofiber membranes. The membranes were observed using a thermal field emission scanning electronic microscope (FE-SEM, JEOL JSM-7800F, Akishima, Tokyo, Japan), and the diameter of the nanofiber membrane was measured by ImageJ software Version 1.53n (NIH, Bethesda, MD, USA) [38].

### 2.6. Preparation of Dextran Polyaldehyde Crosslinker

Dextran polyaldehyde crosslinker was produced based on Hong et al. [38]. A total of 1 g dextran and 2.3 g sodium metaperiodate were dissolved in 30 mL sodium phosphate buffer (50 mM, pH 6.0) and oxidized in the dark for 2 h. The reaction was terminated by adding 100 μL of ethylene glycol followed by dialyzing in sodium phosphate buffer overnight. The dextran polyaldehyde crosslinker was kept at 4 °C, in a refrigerator, until use.

### 2.7. Immobilized BoPAL3 Protein on Nanofiber Membrane by Crosslinking

A total of 3 mg nanofiber membrane was crosslinked with purified recombinant BoPAL3 protein, with vigorous shaking at 150 rpm for 10 min. To optimize the immobilization parameters, dextran polyaldehyde crosslinker percentages of 1 to 5% and incubation times of 3 to 24 h were examined. The BoPAL3/PVA/nylon 6/CS nanofiber membrane was rinsed with sodium phosphate buffer before its residual PAL activity was analyzed.

### 2.8. Biochemical Properties Analysis of Free and Immobilized BoPAL3 Proteins

The protein concentration was analyzed using the protein–dye binding assay [6,7,8], with bovine serum albumin (BSA) as the reference. PAL activity was assayed by the synthesis of *trans*-cinnamic acid, with increased absorbance at the wavelength of 290 nm [6,7,8]. A total of 50 mM Tris–HCl (pH 8.5), 12.1 mM L-phenylalanine, and an aliquot of recombinant BoPAL3 protein were used as the ingredients in the reaction mixture, which had a total volume of 1.0 mL. After 30 min incubation at 37 °C, the assay mixture was stopped by adding 100 μL of 6N HCl [9,10]. D-phenylalanine was used as the blank substrate in the parallel assay. The *trans*-cinnamic acid produced in micromoles per minute was the definition of one PAL activity unit (U). To optimize the reaction temperature, PAL activity assays were carried out at standard reaction conditions in a range of temperatures between 25 and 70 °C. To optimize the reaction pH, PAL activity assays were carried out at 37 °C for 30 min using a universal buffer (40 mM acetic acid, 40 mM boric acid, and 40 mM phosphoric acid) in a pH range from 5 to 11. The maximum PAL activity at optimum conditions was normalized to 100%.

L-phenylalanine in concentrations between 0.12 and 12.1 mM was utilized in order to determine the kinetic characteristics of the BoPAL3 enzyme. A substrate saturation plot was performed with a 10 min incubation time at 37 °C [9,47]. The Michaelis–Menten equation [50] and double reciprocal plot [51] were employed to compute the *K*_m_, V_max_, and *k*_cat_ values.

### 2.9. Determination of Reusability and Denaturant Tolerance of Immobilized BoPAL3 Protein

The reusability of immobilized BoPAL3 proteins on the nanofiber membranes was determined under standard PAL activity assay conditions. Reactions were performed for 30 min at 37 °C using 12.1 mM phenylalanine as the substrate. The immobilized BoPAL3 protein was resuspended in a new substrate mixture after each cycle in order to measure its residual activity over the course of six cycles.

In denaturant tolerance experiments, PAL activities were compared between free and immobilized BoPAL3 proteins, with or without chemical denaturant. Activity assays were carried out using the standard PAL reaction mixture in the presence or absence of 6 M urea, 2% SDS, or 40% ethanol. PAL activity of the free or immobilized BoPAL proteins without chemical denaturant was measured and normalized as 100%.

### 2.10. Data Statistical Analysis

All experiments were conducted individually in triplicate and data are expressed as mean ± standard deviation (SD, error bars). Graphs and statistical analysis, one-way analysis of variances (ANOVA), were obtained by using SigmaPlot software version 11.0 (Systat Software Inc., San Jose, CA, USA).

## 3. Results and Discussion

### 3.1. Preparation of Recombinant BoPAL3 Protein by Affinity Chromatography

**Figure 2:**
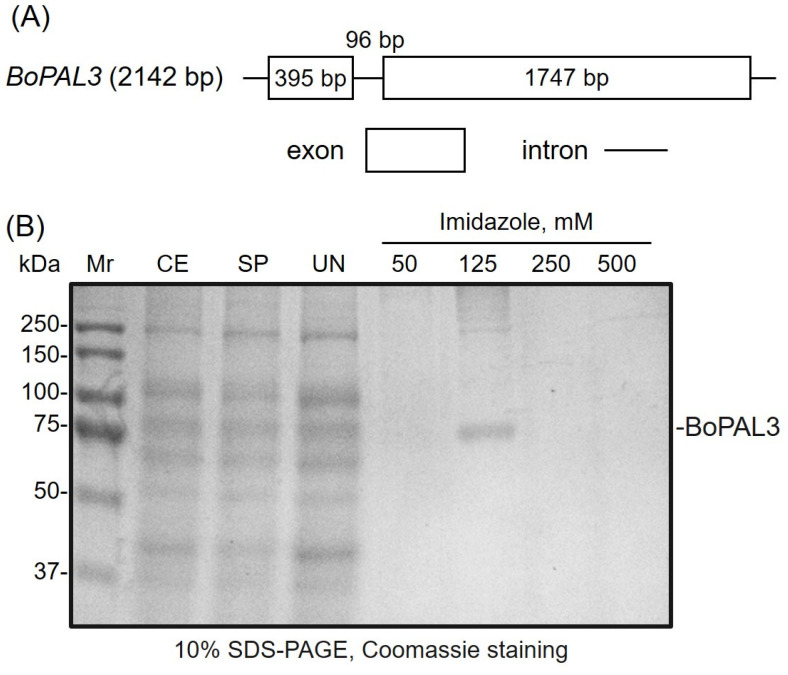
Preparation of recombinant BoPAL3 protein. (**A**) Exons (rectangle) and intron (line) in the *BoPAL3* gene are shown to scale and the number of base pairs (bp) is indicated. (**B**) Ni-NTA resin was used to affinity-purify the N-terminal His-tagged recombinant BoPAL3 protein, which was then separated on a 10% SDS-polyacrylamide gel and stained with Coomassie Brilliant Blue G-250. Mr, Bio-Rad precision plus protein standards; CE, crude extract; SP, soluble protein; UN, unbound protein. Recombinant BoPAL3 proteins that were bound to Ni-resin were eluted by various imidazole buffers, at the indicated concentrations.

The *BoPAL3* gene was isolated using a PCR-based cloning method and deposited in the GenBank^®^ database (NIH, Bethesda, MD, USA) with the accession number: HM009319 [6]. Two exons (2142 bp open-reading frame) in the *BoPAL3* gene were separated by one 96 bp intron and encoded a 713 amino acid polypeptide (Figure 2A). The full-length *BoPAL3* gene was subcloned into the pTrcHisA plasmid and expressed in the *Escherichia coli* Top10 strain [3]. The recombinant BoPAL3 protein was affinity-purified and competitively eluted using a range of imidazole buffers (Figure 2B). In the 125 mM imidazole buffer treatment, the BoPAL3 recombinant protein was isolated to almost homogeneity with a molecular mass of about 75 kDa (Figure 2B). The purification result in this work was comparable to a previous report [3]. The *E. coli* expressed the BoPAL3 protein as an active PAL enzyme, which was, then, used to study the parameters of the enzyme immobilization on the electrospun nanofiber membranes.

### 3.2. Crosslinking Condition Optimization

For electrospinning polyvinyl alcohol (PVA)/nylon 6/chitosan (CS) nanofiber membranes, the ideal flow rate, applied voltage, and distance from spinneret to collector were 0.10 mL/h, 13.8 kV, and 13 cm, respectively [38]. In this study, we continued to follow the standard conditions to obtain electrospun nanofiber membranes, and the diameter of the nanofiber membrane was approximately 120 nm, which was comparable to a previous study [38].

**Figure 3:**
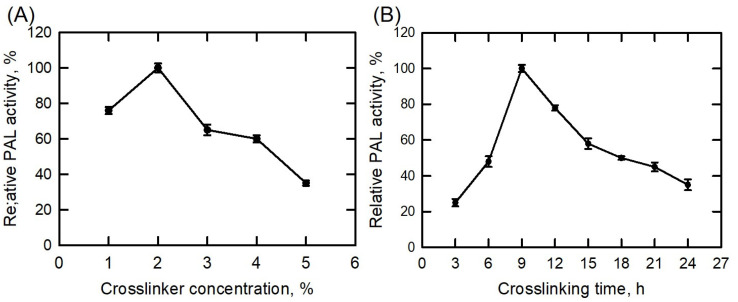
Optimal conditions for crosslinking BoPAL3 proteins on electrospun nanofiber membranes. (**A**) Dextran polyaldehyde concentrations ranging from 1% to 5% were combined with recombinant BoPAL3 protein for 3 h, and then, the PAL enzymatic assay was performed under standard conditions. (**B**) Dextran polyaldehyde of 2% was combined with recombinant BoPAL3 protein for 3 to 24 h, and then, the PAL enzymatic assay was performed under standard conditions. The highest PAL activity by the immobilized BoPAL3 protein was normalized to 100%. Experiments were conducted independently in triplicate, and data are expressed as mean ± standard deviation (S.D.; error bars).

Polysaccharides are common polymeric compounds in nature and have many positive aspects, such as biocompatibility, biodegradability, and so on. One of the major applications of polysaccharides is three-dimensional (3D) crosslinking, which is used in life or pharmaceutical science instead of using a chemical crosslinker, such as disuccinimidyl suberate (DSS), glutaraldehyde, and so on [38,52]. Dextran polyaldehyde was successfully used to crosslink the pectinase enzyme onto the surface of chitosan nanoparticles [53]. As a macromolecule crosslinking agent, dextran polyaldehyde was created and utilized to encapsulate BoPAL3 recombinant protein in the PVA/nylon 6/CS nanofibrous membrane. The hydroxyl groups in dextran were oxidized by sodium periodate to yield dialdehyde groups (at C-2 and C-3 positions of glucose), crosslinking the amine groups on the BoPAL3 protein in the presence of chitosan [38,53]. Recently, chitosan has become a popular material for fabricating membranes [54,55]; however, in this study, chitosan was used as an amine donor to enhance the crosslinking reaction. To optimize the dextran polyaldehyde concentration, the PAL activity was determined at a crosslinker range of 1% to 5%, with the maximum PAL activity of the immobilized BoPAL3 protein measured using the 2% crosslinker treatment (Figure 3A). A decrease in enzyme activities was detected at higher crosslinker concentration (3–5%) treatments, indicating that over-crosslinking may impair the protein structure of the BoPAL3 enzyme as well as its enzyme function. Similar results were observed and published previously [38,53]. To optimize the dextran polyaldehyde crosslinking time, the PAL activity was measured from 3 to 24 h, and the optimum activity of immobilized BoPAL3 protein was detected after the 9 h treatment (Figure 3B). Consistently, an increase in the treatment period may cause over-crosslinking, leading to a reduction in enzyme activity [56,57]. As a result, 2% dextran polyaldehyde combined with a 9 h crosslinking treatment was appropriate for immobilizing the BoPAL3 protein onto the PVA/nylon 6/CS nanofibrous membranes.

### 3.3. Temperature and pH Stability of Free and Immobilized BoPAL3 Proteins

**Figure 4:**
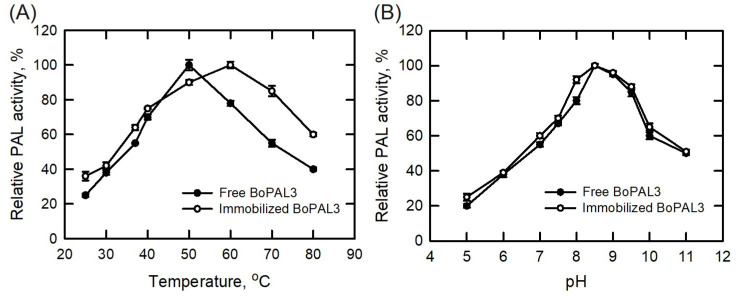
The optimal reaction conditions for free and immobilized BoPAL3 proteins. (**A**) In a temperature range of 25 to 80 °C, the PAL activity of free or immobilized BoPAL3 protein was determined under normal assay conditions. (**B**) Under normal test conditions, the PAL activity of free or immobilized BoPAL3 protein was assessed over the pH range of 5 to 11. The highest PAL activity by the free or immobilized BoPAL3 protein was normalized to 100%. Experiments were performed independently and in triplicate, and data are expressed as mean ± S.D. (error bars).

The optimum temperature of the free BoPAL3 enzyme was 50 °C [3]. In this study, PAL enzyme assays were conducted at temperatures ranging from 25 to 80 °C in order to optimize the reaction temperature for the free and immobilized BoPAL3 proteins (Figure 4A). The BoPAL3 enzyme performed well at temperatures between 50 °C and 60 °C, when it was free and immobilized, respectively (Figure 4A). The immobilized BoPAL3 protein showed better temperature stability than the free BoPAL3 protein, suggesting that the BoPAL3 protein can be stabilized and function at higher temperatures through protein immobilization, which was comparable to a previous study [38].

The optimum pH for the free BoPAL3 enzyme was 8.5 [3]. In this study, PAL enzyme assays were conducted at a pH range of 5 to 11, in order to optimize the reaction pH for the free and immobilized BoPAL3 proteins (Figure 4B). Both the free and immobilized BoPAL3 proteins performed best at pH 8.5 (Figure 4B), indicating that protein immobilization has no effect on pH, which was comparable to a previous study [38].

### 3.4. Kinetic Parameters of Free and Immobilized BoPAL3 Proteins

To study the binding affinity between the substrate and enzyme, the *K*_m_ value was obtained from the substrate saturation curve based on the Michaelis–Menten equation [50], and from the double reciprocal plot based on the Lineweaver–Burk equation [51]. The lower the *K*_m_ value, the higher the affinity toward its substrate. The *k*_cat_ value is the turnover number, which represents the product mole number per enzyme mole number per second. The higher the *k*_cat_ value, the better the catalytic activity. Therefore, the *k*_cat_/*K*_m_ value represents the overall catalytic efficacy of the enzyme.

The *k*_cat_ values of the free and immobilized BoPAL3 enzymes were 1.09 s^−1^ and 1.28 s^−1^, respectively, which were similar to the BoPAL1 enzyme and were lower than for the BoPAL2 enzyme (Table 1). The *K*_m_ values of the free and immobilized BoPAL3 enzymes were 200 μM and 268 μM, respectively, which were lower than for the BoPAL1 and BoPAL2 enzymes (Table 1). The *K*_m_ values of the immobilized BoPAL1–3 proteins were marginally greater than the values for the free BoPAL1–3 proteins, presumably due to the crosslinking effects that limit the permeability of the matrix [38,58,59,60]. This overall kinetic parameter for the free BoPAL proteins was comparable with a previous study [3].

The calculated *k*_cat_/*K*_m_ values for the free and immobilized BoPAL3 proteins were 5.45 × 10^−3^ s^−1^ μM^−1^ and 5.29 × 10^−3^ s^−1^ μM^−1^, respectively (Table 1). In terms of the catalytic property estimated by the *k*_cat_/*K*_m_ value, the free and immobilized BoPAL3 proteins were ranked second among the three BoPAL proteins (Table 1). As a result, similar *k*_cat_/*K*_m_ values were measured for both the free and immobilized BoPAL proteins. This suggests that through the effects of crosslinking, BoPAL proteins do not significantly reduce catalytic effectiveness and specificity.

### 3.5. Reusability and Storage Stability of Free and Immobilized BoPAL3 Proteins

**Figure 5:**
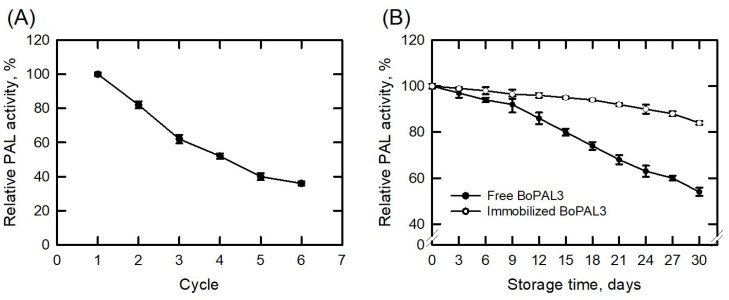
Properties of immobilized BoPAL3 protein. (**A**) Immobilized BoPAL3 activity was assessed under standard assay conditions on electrospun nanofiber membranes for 30 min. Afterward, it was transferred to the subsequent reaction mixture and tested again, six times in total. (**B**) The storage integrity of free and immobilized BoPAL3 proteins was monitored at 4 °C and PAL activity was measured under standard assay conditions every 3 days up to 30 days. The highest PAL activity of the free or immobilized BoPAL3 protein was normalized to 100%. Experiments were performed independently and in triplicate, and data are expressed as mean ± S.D. (error bars).

The recyclability of immobilized enzymes is one of the important factors in industrial applications. PAL activity was measured under standard reaction conditions at pH 8.5 with 12.1 mM Phe for 30 min. After six consecutive cycles of PAL activity measurements, the immobilized BoPAL3 protein still had 36% residual activity (Figure 5A), indicating that the BoPAL3/PVA/nylon 6/CS nanofibrous membrane was capable of being reused.

The free and immobilized BoPAL3 proteins were kept at 4 °C to assess their storage stability, and after 3 to 30 days, the PAL activity was determined under standard reaction conditions at pH 8.5 with 12.1 mM Phe for 30 min (Figure 5B). The residual activity of the free BoPAL3 protein was 56%, while the immobilized BoPAL3 protein retained 84% residual activity. The enhanced storage stability of the immobilized BoPAL3 protein may be, presumably, due to the crosslinking to the BoPAL3/PVA/nylon 6/CS nanofibrous membrane, preventing the potential strain effects at the active site [38,53]. Thus, immobilization of the BoPAL3 protein on the membrane can prolong its stability in long-term cold storage conditions.

### 3.6. Denaturant Tolerance Assay between Free and Immobilized BoPAL3 Proteins

**Figure 6:**
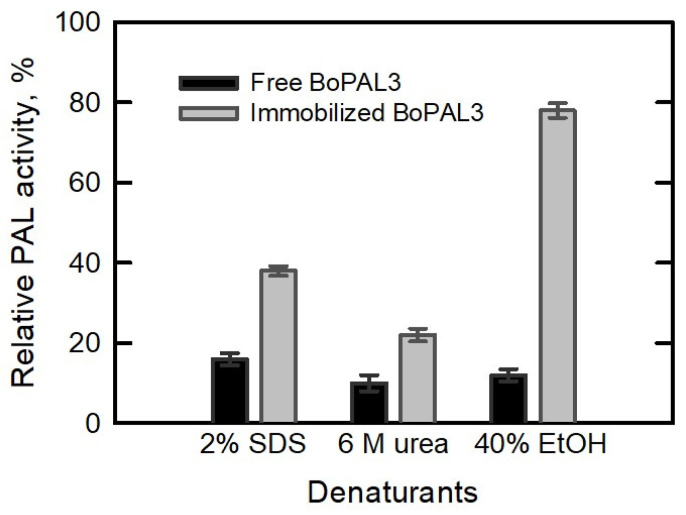
Denaturant tolerance assay of free and immobilized BoPAL3 proteins. Relative PAL activity of free or immobilized BoPAL3 proteins was performed under standard assay conditions with 2% SDS, 6 M urea, or 40% ethanol. The enzymatic activity of free or immobilized BoPAL3 proteins in the absence of denaturant was normalized to 100%. Experiments were performed independently and in triplicate, and data are expressed as mean ± S.D. (error bars).

Enhancement of denaturant tolerance is another positive aspect of protein immobilization applications [38,59,60]. To compare denaturation resistance, the residual activity of the free and immobilized BoPAL3 proteins was approximately 16% and 38%, respectively, for 6 M urea-treated moieties, 10% and 22%, respectively, for 2% SDS-treated moieties, and 12% and 78%, respectively, for 40% ethanol-treated moieties (Figure 6). This result indicates that BoPAL3/PVA/nylon 6/CS nanofibrous membranes are relatively resistant to these three chaotropic chemicals [38,59,60].

## 4. Conclusions

Recombinant BoPAL3 protein was purified and immobilized on electrospun nanofiber membranes using dextran polyaldehyde as a natural/polymeric crosslinker to produce *trans*-cinnamic acid. The resulting immobilized BoPAL3 protein showed superior thermal stability at 60 °C, catalytic activity, reusability, denaturation resistance, and storage stability for 30 days over the free BoPAL3 protein. In addition, 2% dextran polyaldehyde treatment for 9 h was the best condition to conjugate BoPAL3 protein onto the PVA/nylon 6/CS membrane. Overall, this immobilization technology uses biocompatible natural materials, has serious environmental benefits, and increases the utility of BoPAL proteins in comparison to typical chemical crosslinking reagents.

## Figures and Tables

**Table 1:** Kinetic comparison between free and immobilized BoPAL1–3 proteins.

Enzymes	*k*_cat_ (s^−1^)		*K*_m_ (µM)		*k*_cat_/*K*_m_ (s^−1^ µM^−1^)		Reference
	Free	Immobilized	Free	Immobilized	Free	Immobilized	
BoPAL1	1.01	1.21	518	534	1.91 × 10^−3^	2.2 × 10^−3^	[38]
BoPAL2	4.02	3.99	329	379	1.23 × 10^−2^	1.05 × 10^−2^	[38]
BoPAL3	1.09	1.28	200	268	5.45 × 10^−3^	5.29 × 10^−3^	This study

## Data Availability

Data is included in the article.
